# Ileo-ileal intussusception caused by small bowel leiomyosarcoma: A rare case report

**DOI:** 10.1016/j.ijscr.2020.05.049

**Published:** 2020-05-29

**Authors:** Erica Mazzotta, Sara Lauricella, Filippo Carannante, Gianluca Mascianà, Marco Caricato, Gabriella T. Capolupo

**Affiliations:** Colorectal Surgery Unit, Università Campus Bio-Medico, Rome, Italy

**Keywords:** Intussusception, Leiomyosarcoma, Ileal neoplasm

## Abstract

**Introduction:**

Intussusception is the telescoping of one segment of the bowel into an adjacent bowel segment, causing venous congestion, edema, and blood supply reduction. We present a case of ileo-ileal intussusception in an adult patient with intestinal obstruction caused by a rare mesenchymal malignant lesion of the distal ileum: Leiomyosarcoma (LMS).

**Presentation of case:**

A 90-year-old Caucasian man presented to the hospital with a two-day history of abdominal pain, nausea, and bowel occlusion. Preoperative Computer Tomography (CT) showed a solid mass with stratified walls in the lumen of the cecum with the classics “bulls-eye” appearance with concentric rings, suggestive of intussusception. The patient underwent emergency laparotomy with evidence of a small bowel wall tumor driving ileo-ileal intussusception with ischemic damage. Ileocecal resection was performed without postoperative complications. Histopathological examination showed a tumor on the muscular layer of the small bowel. The definitive diagnosis was LMS.

**Discussion:**

Adult intussusception is a rare condition, with an incidence of 2/1 000 000 cases per year worldwide. About 60% of patients suffering from this disease require surgery. Clinical presentation can be non-specific because of its no characteristic signs and symptoms. The most common presenting symptom is abdominal pain with bowel obstruction sings. Intussusception can occur anywhere along the small and large intestine and it is typically associated with a Lead Point (LP). The LP may be benign or malignant conditions. Infrequent malignant causes include LMS.

**Conclusion:**

Diagnosis of intussusception is relatively challenging because of its non-specific symptoms. CT scan is the examimation of choice for the diagnosis because of its peculiar images. In adults, surgical treatment is recommended with laparoscopic or open approach according to surgeon expertise, sometimes in an emergency setting.

## Highlights

• Here we reported a rare case of ileo-ileal intussusception caused by small bowel leiomyosarcoma.
• Actually the case reported in literature of ileo-ileal intussusception are rare and mechanism is still unclear.
• The cases of leiomyosarcoma present in the literature are few and even rarer those in adults.

## 1 Introduction

Intussusception is the invagination of a bowel loop with its mesenteric fold into the lumen of an adjacent part of the bowel. This condition limits the venous drainage of the mesentery, leading to venous congestion and tissue edema, compromising peristalsis, and regular intestinal transit. If untreated, bowel ischemia, necrosis, and finally perforation can follow.

Intussusceptions can be with or without Lead Point (LP). Intussusception without LP frequently occurs in children or adults with celiac disease and Crohn disease [1]. It tends to be transient without mechanical bowel obstruction and could be treated conservatively [2]. Intussusception with LP is caused by an underlying neoplasm, benign or malign, frequently presenting with bowel obstruction and acute abdomen. In these cases, surgical management is required, often in an emergency setting.

We present a case of ileo-ileal intussusception in an adult patient with intestinal obstruction caused by a rare mesenchymal malignant lesion of the distal ileum: Leiomyosarcoma (LMS).

This work has been reported in line with the SCARE criteria 2018 [3].

## 2 Presentation of case

Written informed consent was obtained from the patient for publication of this case report and any accompanying images.

**Fig. 1:**
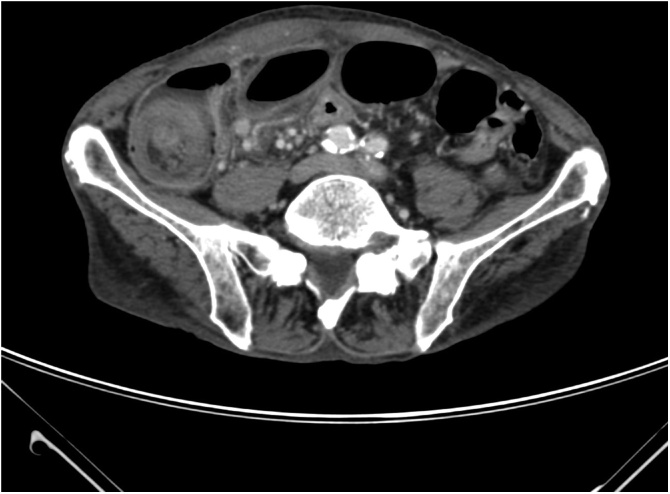
Classic “Bulls eye” appearance with concentric rings in a CT scan axial view; evidence of solid mass of the terminal ileum with stratified walls in the lumen.

A 90-year-old Caucasian man was admitted with a two-day history of abdominal pain, nausea, and occlusion. He had hypertension and dyslipidemia under treatment. No drug allergy was reported. His past surgical history included a right open inguinal hernia repair and hemorrhoidectomy. Physical examination showed distended and tympanic abdomen with tenderness in the right lower quadrant. No palpable mass in the abdomen was noted. Laboratory tests revealed WBC 6.9 10^3^/uL, RBC 3.46 10^6^/uL, PLT 254 10^3^/μL, and low-grade anemia Hb 9.3 g/dL. The CEA and CA19.9 levels were normal. Chest radiography was normal without any evidence of parenchymal lesions or inflammation. Abdomen x-rays showed air-fluid levels without subdiaphragmatic free gas. Colonoscopy until splenic flexure did not reveal any pathologic lesions. Preoperative Computer Tomography (CT) showed a solid mass with stratified walls in the lumen of the cecum with the classics “bulls-eye” appearance with concentric rings, suggestive of ileocolic intussusception (Fig. 1). Moreover, CT demonstrated small bowel wall ischemia and venous congestion of the ileum for 20 cm proximal to the ileocecal valve. A mesenteric lymphadenopathy (max 13 mm diameter) was described in the right mesocolic tissue.

**Fig. 2:**
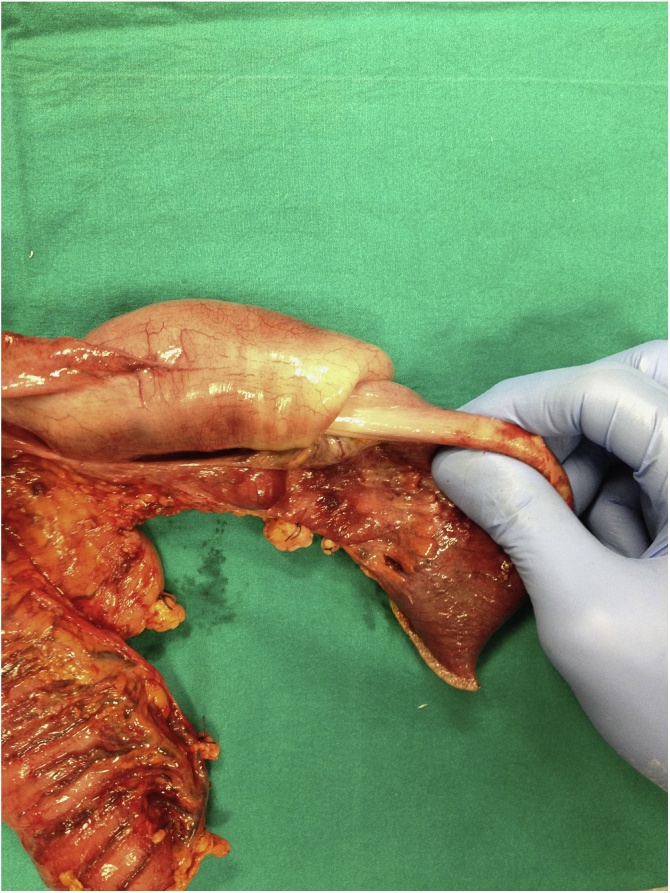
Characteristic “telescoping” of the small bowel.

**Fig. 3:**
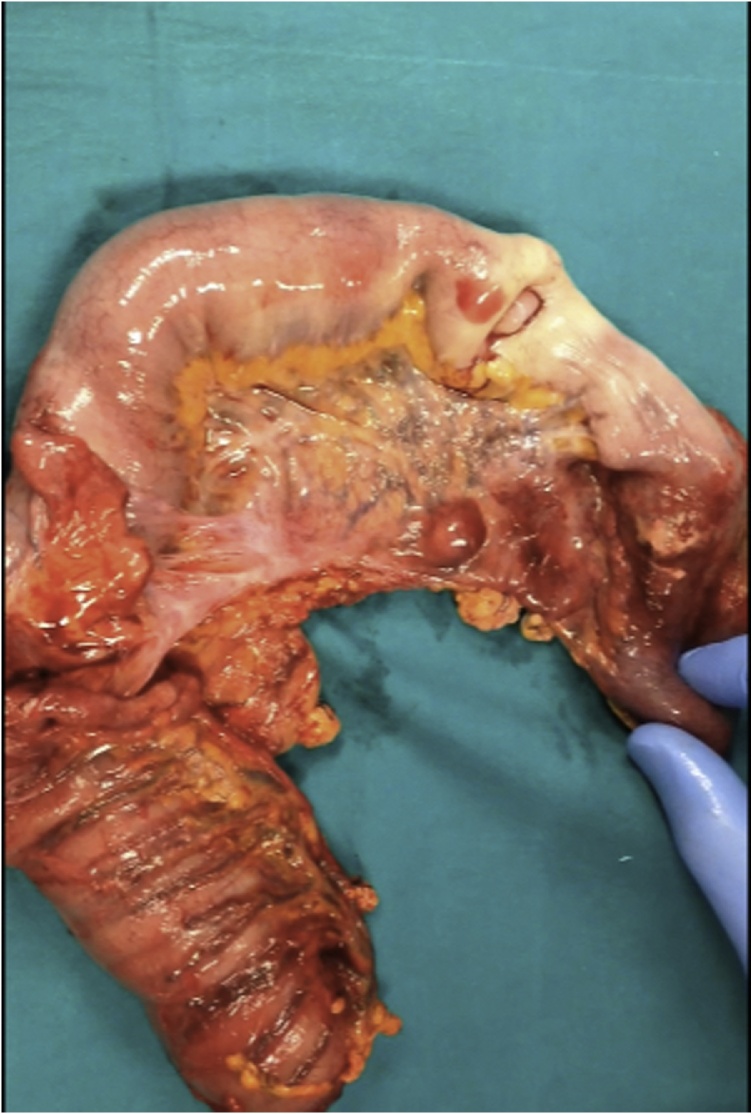
Ileal leiomyosarcoma with ischemic suffering of the adjacent intestinal bowel wall.

Explorative laparotomy was performed by the head physician of our Colorectal Surgery Unit (M. C.). Despite CT findings, we found only an ileal palpable mass causing ileo-ileal intussusception, which did not reach the cecum. The small bowel was dilated, and enlarged nodes were apparent in the mesentery of the distal ileum. Ileocecal resection was performed involving 20 cm of the terminal ileum and palpable mesenteric lymphadenopathy, using two firings of a GIA 60 stapler. A manual latero-lateral isoperistaltic ileocolic anastomosis was performed. Macroscopic examination showed ileo-ileal intussusception lead by a solid ileal mass located 5 cm proximal to the ileocecal valve (Fig. 2). About 5 cm of ileum proximal to the intussusception showed bowel wall ischemia, due to compression of the mesentery (Fig. 3). The postoperative course was regular without complications. The patient noted a decrease in abdominal pain and nausea from early postoperative hours. Twenty-four hours after the operation, he started to eat with good tolerance. The patient was discharged home on Day 5 with follow-up at 15 and 30 postoperative Days.

Histopathological examination showed a tumor on the muscular layer of the small bowel. Immunohistochemical analyses showed actin and desmin positive reactivity, CD117, CD34, DOG 1, and S100 negativity. The definitive diagnosis was LMS G2 grade FNCLCC. After the oncological evaluation, no more surgical or medical treatment was needed.

## 3 Discussion

Intussusception was firstly described by Paul Barbette [4] in 1674, and the Scottish surgeon James Hunter coined the term “intussusception” in 1793 [5]. Intussusception is the telescoping of one segment of the bowel into an adjacent bowel segment, causing venous congestion, edema, and blood supply reduction. This condition is called “telescoping” because it resembles how a collapsible telescope folds into itself. It can occur anywhere along the small and large intestine. According to the position of the LP, we can identify four categories of intussusceptions: entero-enteric (small bowel only), colo-colic (large bowel only), ileocolic (terminal ileum prolapses within the ascending colon), and ileocecal (ileocecal valve is the LP) [2].

Intussusception in childhood is common in infants between the age of 5 and 18 months, with an incidence from 15 to 300/100 000 children < 1 year of age per year worldwide [6]. It is typically an idiopathic and benign condition, presenting as ileocolic intussusception in most cases. It is the first cause of ‘acute abdomen’ in children and the second most common cause of intestinal obstruction after pyloric stenosis [7]. Conservative therapy can be adopted in hemodynamically and clinically stable children. It is typically managed with nonoperative reduction via pneumatic and/or hydrostatic enemas. Surgical treatment is indicated when bowel necrosis, bowel perforation or peritonitis are suspected or evident. Adults intussusceptions are rare, with an incidence of 2/1 000 000 cases per year worldwide [8]. The mean age of intussusception in adults is 50 years with no gender predominance. In about 90% of cases, intestinal intussusceptions have an identifiable etiology [9]. Most adult intussusceptions arise from the small bowel, and the LP is usually a benign condition such as adhesions, strictures, Meckel's diverticulum, inflammatory bowel disease, and benign tumors (lipomas and leiomyomas) [8,10,11]. Sometimes, the LP may be a malignant lesion, most commonly metastatic lesions (from melanoma, breast, and lung), colonic adenocarcinoma and lymphoma. Other infrequent malignant causes associated with small bowel intussusception include gastrointestinal stromal tumors (GISTs), malignant fibrous histiocytomas, carcinoid tumors, neuroendocrine neoplasms, and leiomyosarcomas (LMSs) [12].

The clinical presentation can be non-specific because of its no characteristic signs and symptoms. Intussusception is the cause of 1%–5% of bowel obstruction cases in adults [13]. The most common presenting symptom is abdominal pain with bowel obstruction sings. Other frequent symptoms are abdominal mass, fever, bowel perforation, and gastrointestinal (GI) bleeding [14]. Symptoms presentation is typically acute, with longer onset in large bowel than in small intestine [15]. Laboratory tests usually reveal an elevated white blood cell count and nonspecific inflammatory markers increase, such as high C-reactive protein. Radiological images help in the differential diagnosis. Ultrasound is the exam of choice in diagnosing intussusception in children with elevated sensitivity and specificity [16]. In adults, the choice of imaging modality is essential for a timely diagnosis.

Computed Tomography (CT) scan is the examination of choice for the diagnosis of intussusception with high diagnostic accuracy. The peculiar image on CT is described as “bulls-eye” or “sausage-shaped” lesions appearance with concentric double-ring seen at the axial and coronal view [17] (Fig. 1). Additionally, CT scan may also show mesenteric vessels, bowel wall, and vascular perfusion and may also define the location and the nature of the mass, its relationship to surrounding tissues. Colonoscopy is another useful tool in evaluating intussusception, mainly when the presenting symptoms include a large bowel obstruction.

Intussusception must be identified and carefully treated. Reduction via pneumatic and/or hydrostatic enemas in children is effective, and surgical treatment is reserved only in selected critical cases. Adults intussusception is typically associated with pathological LP; therefore, surgical treatment is recommended, with laparoscopic or open approach according to surgeon expertise. When bowel ischemia, necrosis or perforation is suspected, immediate surgery is required.

In this paper we report a case of ileo-ileal intussusception due to a rare neoplastic malignant lesion of mesenchymal origin in the preterminal ileum: LMS.

Small bowel constitutes 75% of the length of the GI tract. Nevertheless, the incidence of small intestine cancers accounts for less than 5% of all GI cancer cases [18]. About 10–20% of small intestine malignant tumors are LMSs [19]. They most commonly originate in the retroperitoneal space, uterus, vascular wall, and soft tissues. Primary GI LMS is rare (incidence of 0,3–1% [20]), arising mostly in the esophagus, stomach, and small bowel. Terminal ileus is the second most common site of the tumor in the small intestine following duodenum. Abdominal pain and GI bleeding are the most frequent symptoms of GI LMS. Surgical resection with negative margins is the gold standard of treatment. Morphologically, LMS is a smooth muscle cell (SMC) malignant neoplasm with high mitotic counts, necrosis, and cytological atypia at histopathological examination [21].

Before the discovery of Immunohistochemical (IHC) profile, most GI LMSs were classified as GISTs. Definitive tools to differentiate LMSs from GISTs were introduced in the late 1990s [21,22]. These two pathological entities are different in its Immunohistochemical (IHC) and clinicopathological profile: LMSs are positive for smooth muscle cell markers (smooth muscle actin, desmin, caldesmon) and negative for tyrosine kinase c-kit (CD117) and CD 34; GISTs are often CD34 immunoreactive and express CD117 receptor activity. However, 4–5% of GISTs might be negative for CD117. As for the pathological characteristics, GISTs arise from the interstitial cells of Cajal, and the cellular morphology ranges from spindle-shaped cells to epithelioid. In contrast, LMSs originate from SMCs of the muscularis mucosa or propria, and are usually composed of elongated cells with abundant cytoplasm [[23], [24], [25]].

Many papers confirm the rarity of intussusception caused by adults' small bowel LMS. In a PubMed search using the keywords “intussusception” “leiomyosarcoma”, we found 21 case reports about GI intussusception caused by LMS, both in adult and pediatric patients (17 vs 4). Only 5 cases occurred in the terminal ileum of adults [12,21,[26], [27], [28]], as in our patient. This paper confirms the unusual location of LMS along the GI tract and presents current knowledge about etiology, diagnosis and treatment of this rare disease.

## 4 Conclusions

This paper presents an unusual location of LMS along the GI tract causing ileo-ileal intussusception. Due to its non-specific symptoms, the diagnosis was made with preoperative CT scan together with intraoperative findings and histological examination. Surgical management is the treatment of choice in similar conditions, sometimes in an emergency setting.

## Declaration of Competing Interest

All authors disclose any financial and personal relationships with other people or organisations.

## Sources of funding

No sources of funding was used for this research.

## Ethical approval

This study is exempt from ethnical approval in our institution.

## Consent

Written informed consent was obtained from the patient for publication of this case report and accompanying images. A copy of the written consent is available for review by the Editor-in-Chief of this journal on request.

## Author contribution

E. Mazzotta MD – Lauricella Sara MD – F. Carannante MD – G. Mascianà MD – G.T. Capolupo MD PhD FACS: Patient care and management; image contribution.

M. Caricato MD PhD FACS: revision and final approval of the manuscript.

## Registration of research studies

None.

## Guarantor

M. Caricato MD, FACS.

## Provenance and peer review

Not commissioned, externally peer-reviewed.
